# Bushen-Huoxue-Mingmu-Formula attenuates pressurization-induced retinal ganglion cell damage by reducing mitochondrial autophagy through the inhibition of the Pink1/Parkin pathway

**DOI:** 10.1097/MD.0000000000041257

**Published:** 2025-01-10

**Authors:** Wei Wang, Jia Gao, Qianqian Mu, Dan Zhang, Fen Yang, Wubo Cheng

**Affiliations:** a Opthalmology, Kunming Municipal Hospital of Traditional Chinese Medicine, Kunming, China; b Chengdu University of Traditional Chinese Medicine, Chengdu, China; c Yunnan University of Chinese Medicine, Kunming, China; d Outpatient Office, Kunming Municipal Hospital of Traditional Chinese Medicine, Kunming, China; e Opthalmology, Chongqing Hechuan District People’s Hospital, Chongqing, China.

**Keywords:** Bushen-Huoxue-Mingmu-Formula, glaucoma, mitochondrial autophagy, Pink1/Parkin, retinal ganglion cells

## Abstract

**Background:**

Bushen-Huoxue-Mingmu-Formula (MMF) has achieved definite clinical efficacy. However, its mechanism is still unclear.

**Objective:**

Investigating the molecular mechanism of MMF to protect retinal ganglion cells (RGCs).

**Methods:**

This study developed a pressurization-induced model of damaged RGCs, which were then treated with a serum supplemented with MMF. The effects of MMF on proliferation, apoptosis, adenosine 5′-triphosphate content, and mitochondrial structure of RGCs were investigated, and the underlying molecular mechanism was explored by RNA interference experiment.

**Results:**

In the pressurization-induced RGC injury model, apoptosis rate increased, cell proliferation decreased, adenosine 5′-triphosphate content reduced, mitochondrial structure was disrupted, BCL2-associated X, cleaved caspase-3, and microtubule-associated proteins light chain 3 II/I protein expression enhanced, B cell lymphoma-2 and p62 protein expression decreased, and the Pink1/Parkin pathway was activated. The stress-induced damage to RGCs was, however, reversible following MMF-mediated inhibition of the Pink1/Parkin pathway. Pink1 short-hairpin RNA downregulated Pink1 expression in RGCs, which led to outcomes that aligned with those observed with MMF intervention.

**Conclusions:**

MMF altered the expression of apoptosis- and autophagy-related proteins and possibly inhibited the Pink1/Parkin signaling pathway, which led to reduced pressurization-induced mitochondrial autophagy in RGCs. This preventive effect of MMF on RGCs can be potentially useful to preserve the viability of RGCs.

## Graphical abstract

**Figure:**
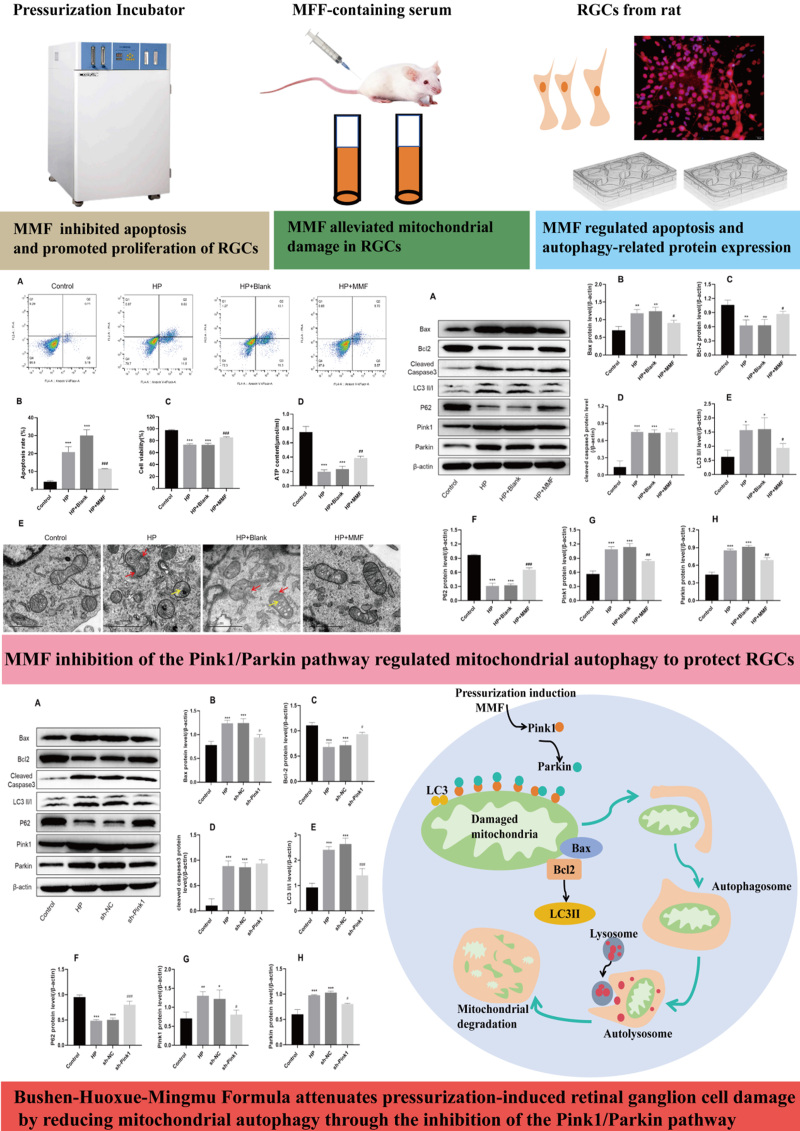

## 1. Introduction

Glaucoma is a leading cause of irreversible blindness. According to the World Health Organization report, 76 million people suffered from glaucoma in 2020.^[1]^ This number is projected to exceed 11.1 million by 2040. Glaucoma, which adversely affects the quality of life of patients is characterized by the selective and irreversible loss of retinal ganglion cells (RGCs) that ultimately leads to visual defects.^[2]^ Injury to RGCs is caused by multiple factors, including elevated intraocular pressure (IOP), ischemia/reperfusion, oxidative stress, neurotrophic growth factor deprivation, and glutamate neurotoxicity.^[3]^ Pathologically, IOP constitutes the primary cause of RGC damage in patients with glaucoma. RGCs are the only retinal efferent neurons that project axons directly into the central nervous system and conduct visual functions.^[4]^ At present, the sole scientifically proven treatment to decelerate or partially prevent glaucoma progression is IOP lowering, which however may fail to completely prevent RGC apoptosis and optic nerve damage. Given that optic nerve preservation is vital to glaucoma treatment,^[5]^ neuroprotective agents such as nitric oxide synthase inhibitors, ion channel blockers, and neurotrophic factors have been employed to for protection of retinal nerve cells. However, these agents suffer from limitations such as difficulty in development and inherent side-effects.^[6]^

Chinese medicine comprises multi-components that aim multiple targets to mediate overall regulation and provide unique advantages in the treatment of glaucoma. As per the five-wheel theory of Chinese medicine, the normal maintenance of the structure and function of the eyes relies on the regulation of the kidneys. Prof Chen Dafu, a famous ophthalmologist in modern Chinese medicine, believes that the optic nerve and retina belong to the Liver Meridian of the Foot-Jueyin and that the liver and kidney have a common origin. Therefore, glaucoma is closely associated with the liver and kidney. Based on these theories and experiences, Chinese medicine proposes nourishment of the liver and kidneys to improve blood circulation and eliminate blood stasis to promote nourishment of the blood to the eyes as the basic strategy for prevention and treatment of optic nerve damage in glaucoma.^[3,7]^ Modern pharmacological studies have also revealed that Chinese medicine offers obvious advantages by improving microcirculation, lowering blood viscosity, enhancing the body’s antioxidant capacity, improving the optic nerve axial plasma flow, and alleviating the microcirculation condition of the optic disc in glaucoma.^[8,9]^ Bushen-Huoxue-Mingmu-Formula (MMF) is a remedy researched at our clinic to nourish the liver and kidneys as well as to improve blood circulation. It comprises Dan Shen (*Salvia miltiorrhiza Bunge*), San Qi (*Panax notoginseng*), Gou Qi Zi (*Lycium barbarum L.*), Ju Hua (*Chrysanthemum morifolium Ramat*), Shu Di Huang (*Rehmannia glutinosa* [*Gaertn.*]), Jiu Yu Rou (*Cornus officinalis*), Mu Dan Pi (*Paeonia suffruticosa*), Shan Yao (*Dioscoreae Rhizoma*), Fu Ling (*Poria cocos* [*Schw.*] *Wolf*), and Ze Xie (*Alisma plantago-aquatica L.*). A previous study had shown that MMF can improve visual field, graphic visual evoked potentials, and retinal nerve fiber layer thickness in patients with controlled IOP after primary glaucoma surgery, thus highlighting its protective effect on the optic nerve and visual function in glaucoma. Furthermore, an MMF-containing serum was found to inhibit stress-induced apoptosis in RGCs. However, the specific mechanism of action underlying MMF-mediated inhibition of pressure-induced injury to RGCs is still unclear.

Accumulating evidence has linked mitochondrial dysfunction to glaucomatous degeneration in several glaucoma models.^[10]^ Mitochondrial dysfunction increases the susceptibility of RGCs to other risk factors, including high IOP, light exposure, and vascular insufficiency.^[11]^ Mitochondrial autophagy as a quality control process essentially ensures the right quantity and quality of mitochondria.^[12]^ Studies suggest that impaired mitochondrial autophagy represents a feature of glaucomatous degeneration.^[13,14]^ PTEN-induced kinase 1 (PINK1) and Parkin RBR E3 ubiquitin-protein ligase (Parkin) signaling play key roles in determining mitochondrial mitosis, motility, and size. Any injury/dysfunction can decrease the mitochondrial membrane potential and contribute to the accumulation of PINK1 at the outer mitochondrial membrane. The recruited Parkin in turn translocates from the cytosol to the outer mitochondrial membrane and promotes mitochondrial autophagy via ubiquitination of mitochondrial proteins, which leads to mitochondrial degradation.^[15]^ Mitochondrial autophagy and the Pink1/Parkin pathway have been closely associated with glaucoma progression.^[16]^

This study determined the role of MMF in preventing mitochondrial autophagy related to pressurization-induced death of RGCs through the regulation of the Pink1/Parkin pathway, and investigated the underlying mechanism to provide a theoretical basis for the clinical treatment of glaucoma.

## 2. Methods

### 2.1. Statement of ethics

Twenty specific pathogen-free (SPF)-grade male SD rats, 8 to 10 weeks old and weighing 280 to 300 g, were purchased from Yunnan University Laboratory Animal Center (animal license No. SCXK [Yunnan] K2021-0001). The animal experiment protocol was approved by the Ethical Review Committee for Laboratory Animals of Yunnan Besitel Biotechnology Co., Ltd (Approval No. BST-RAT-20230322-01).

### 2.2. Cell culture

Rat RGCs (RAT-iCell-m027) were obtained from Icellbioscience Technology Co., Ltd., Shanghai and cultivated in a iCell Primary Neuronal Cell Culture System (Icellbioscience) in an incubator at 37 °C with 5% carbon dioxide (CO_2_). The medium was replaced every 2 days, and the growth process and morphological alterations in cells were examined using an inverted phase-contrast microscope (Nikon, Japan).

### 2.3. Pressure-induced damaged model of RGCs

A modified open-pressure controlled culture system was developed by adding a vacuum gas pressure pump to a standard culture setup to facilitate the dynamic circulation of gas between the incubator and culture bottles. The pressure within culture bottles was moderated through the adjustment of inlet and output pressures and flow rates based on pressure gauge readings. The incubator was set at 37 °C and a CO_2_ concentration of 5%. After 48 hours of cell culture, half of the complete medium was replenished and the cells were transferred to the pressure-controlled culture system where they were exposed to varying pressure levels (0, 20, 40, 60, and 80 mm Hg). Cells were harvested for analysis after 24 hours of incubation, and each set of experiments was replicated thrice. Based on the experimental results, the optimal pressure value is selected to be defined as high pressure (HP).

### 2.4. Preparation of MMF-supplemented serum

The MMF Formula was prepared using Compound Danshen Tablet containing Dan Shen and San Qi and Qi Ju Di Huang Wan, which contains Gou Qi Zi, Ju Hua, Shu Di Huang, Jiu Yu Rou, Mu Dan Pi, Shan Yao, Fu Ling, and Ze Xie. Prior to the preparation of the MMF gavage suspension, 4.32 g of Compound Danshen Tablet and 16.875 g of Qi Ju Di Huang Wan were weighed on an electronic balance and pulverized to prepare a powder. The obtained powder was mixed with 45 mL of saline. Twenty SPF-grade male SD rats (6–8-week-old) were randomly divided into 2 groups (control and MMF group), with 10 rats in each group. Humidity and ambient temperature of the rats were maintained between 50% to 70% and 20 °C to 26 °C, respectively. The 2 groups were orally administered with saline or compound suspension (100 g/mL, 7 days, once/day). Two hours post-administration of the last gavage, SD rats treated with MMF-containing serum were weighed and anesthetized. Their blood was respectively collected from the abdominal aorta, refrigerated at 4 °C for overnight, and centrifuged at 800 × g for 15 minutes in a low-speed centrifuge. The serum obtained was collected from the upper layer and inactivated at 56 °C for 30 minutes in a water bath. The same group of sera was mixed to maintain a homogeneous drug concentration in the serum. The serum was then filtered through a 0.22 μm microporous membrane (Millipore, Billerica, MA) and stored at −20 °C.

### 2.5. Pink1 short-hairpin RNA (shRNA) preparation and transfection

RGCs were seeded in six-well plates at a density of 1 × 10^6^ cells/well. Upon reaching 80% confluency, the cells were infected with a Pink1-shRNA lentiviral vector reagent (Generay, Shanghai, China) at a multiplicity of infection of 50. The transfection efficiency was detected after 72 hours of incubation in a cell culture incubator. Sequences of sh-RNAs are shown in Supplementary Material S1, Supplemental Digital Content, http://links.lww.com/MD/O265.

### 2.6. Cell Counting Kit (CCK)-8 assay

RGCs were inoculated in 96-well plates at a density of 5 × 10^3^ cells/well for intervention, and their activity was detected by a CCK-8 assay kit (Solarbio, Beijing, China). In brief, treated cells were incubated with CCK-8 solution (10 μL) for 4 hours, and the absorbance of each well was measured at 450 nm wavelength. The cell activity was calculated based on the absorbance value.

### 2.7. Flow cytometry

Apoptosis of cells was investigated using an Annexin V-APC/propidium iodide apoptosis detection kit (Vazyme, Nanjing, China). Cells were collected and incubated with 10 μL each of Annexin V and propidium iodide at 37 °C for 20 minutes. Following incubation, the samples were mixed with Ending buffer, rinsed thrice for 5 minutes each, and subjected to flow cytometry analysis using a BD Accuri™ C6 Plus flow cytometer (BD Biosciences, Franklin Lakes, NJ) as per the manufacture’s protocol.

### 2.8. Transmission electron microscopy (TEM)

The ultrastructure of the mitochondrion of RGCs was assessed by TEM. Cells were collected and fixed in 2.5% glutaraldehyde for 2 hours at 4 °C, treated with 1% osmium tetroxide for 1 hour at 4 °C, dehydrated, and embedded in epoxy resin. Cells were sectioned into ultra-thin slices (60–80 nm) using an ultrathin microtome (RMC/MTX; Elexience, Verrières-le-Buisson, France). The slices were mounted on a copper grid, contrasted with 8% uranyl acetate and lead citrate, and visualized under a Philips CM100 transmission electron microscope equipped with a TENGRA 2.3K X 2.3K TEM camera. Analysis was performed using a soft imaging system.

### 2.9. Measurement of ATP content

The adenosine 5′-triphosphate (ATP) content of cells was measured using an ATP assay kit (Solarbio, Beijing, China). Test cells were collected and mixed with 1 mL of an extraction solution. The cells were sonicated using ultrasonic waves (ice bath, power 200W, ultrasonic 2 seconds, stop 1 second, total time 1 minute) and centrifuged to obtain a supernatant (10,000 × g, 4 °C, 10 minutes). The supernatant was treated with 500 μL of chloroform, mixed well, and centrifuged at 10,000 × g for 3 minutes at 4 °C. The absorbance of the obtained solution was measured at 340 nm wavelength.

### 2.10. Quantitative real-time polymerase chain reaction

Total RNA from treated cells was extracted using RNA Easy Fast Tissue Kit (TIANGEN, Beijing, China), and its quality and concentration were determined by agarose gel electrophoresis and NanoDrop 2000 (Thermo Fisher Scientific, Wilmington, NC), respectively. The RNA was used for the synthesis of cDNA using HiScript III 1st Strand cDNA Synthesis Kit (+gDNA wiper) (Vazyme, Nanjing, China) following the manufacturer’s protocol.

The expression level of Pink1 was determined by quantitative real-time polymerase chain reaction on an ABI StepOne Plus real-time PCR system (PerkinElmer Applied Biosystems, Foster City, CA) using ChamQ SYBR qPCR Master Mix (Vazyme, Nanjing, China). The expression of Pink1 target gene relative to that of glyceraldehyde 3-phosphate dehydrogenase was calculated by the 2^−ΔΔCt^ method.^[17]^ All reactions were performed in triplicates using primers shown in Table 1.

**Table 1:** Primers used for RT-qPCR.

Name	Sequence (5′–3′)
GAPDH-F	GTGGAGTCTACTGGCGTCTT
GAPDH-R	TGCTGACAATCTTGAGGGA
Pink1-F	AGACGGTCCCAAGCAGCTT
Pink1-R	AAG TGAAGGCGCGGAAAAC

GAPDH = glyceraldehyde-3-phosphate dehydrogenase; Pink1 = PTEN-induced putative kinase 1, qRT-PCR = quantitative real-time polymerase chain reaction.

### 2.11. Western blot analysis

The collected cells were lysed in a radioimmunoprecipitation assay buffer (Beyotime, Shanghai, China), and centrifuged at 4 °C and 10,000 × g for 10 minutes to obtain a protein-rich supernatant. The protein concentration was determined using a bicinchoninic acid assay kit (Ebio-ace, Nanjing, China). The samples were denatured in a water bath at 95 °C for 15 minutes, and then separated on 4% to 20% sodium dodecyl sulfate polyacrylamide gel electrophoresis gels (Ebio-ace, Nanjing, China). The separated protein bands were transferred onto polyvinylidene fluoride membranes, which were then blocked with 5% (w/v) skim milk powder to prevent any nonspecific reactions. The membranes were incubated for overnight at 4 °C with antibodies specific to target proteins. The membranes were washed thrice, and then probed with a horseradish peroxidase-labeled secondary antibody. Protein bands were visualized using a BIO-RAD Gel Doc XR + imaging system (Hercules, CA) and analyzed by ImageJ software (National Institutes of Health, Bethesda, MD) for grayscale values. The sources and dilution ratios of the antibodies are presented in Table 2.

**Table 2:** Sources and dilution ratios of antibodies used for Western blotting.

Name	Sources	Catalog	Dilution ratio
Bax	Huabio, Hanzhou, China	ET1603-34	1:2000
Bcl2	Huabio, Hanzhou, China	ET1702-53	1:2000
Cleaved-caspase3	Abcam, Cambridge, MA	ab214430	1:3000
LC3 II/I	Abcam, Cambridge, MA	ab128025	1:3000
P62	Huabio, Hanzhou, China	HA721171	1:2000
Pink1	Huabio, Hanzhou, China	ER1706-27	1:2000
Parkin	Huabio, Hanzhou, China	RT1461	1:2000
β-actin	Huabio, Hanzhou, China	R1207-1	1:2000

Bax = BCL2-associated X; Bcl2 = B-cell lymphoma-2; LC3 II/I = microtubule-associated protein 1 light chain 3; P62 = sequestosome 1; Pink1 = PTEN-induced putative kinase 1; Parkin = E3 ubiquitin-protein ligase parkin; β-actin = beta-actin.

### 2.12. Statistical analysis

Statistical analyses were performed using the SPSS 26.0 software (version 25.0, Chicago, IL). Data were presented as mean ± standard deviation, and conformed to normality and chi-square. One-way analysis of variance was used for between-group comparisons, and the least significant difference method was employed for multiple comparisons. A value of *P* < .05 indicated significant difference.

## 3. Results

### 3.1. Pressurization of RGCs led to apoptosis and inhibition of proliferation

**Figure 1.**
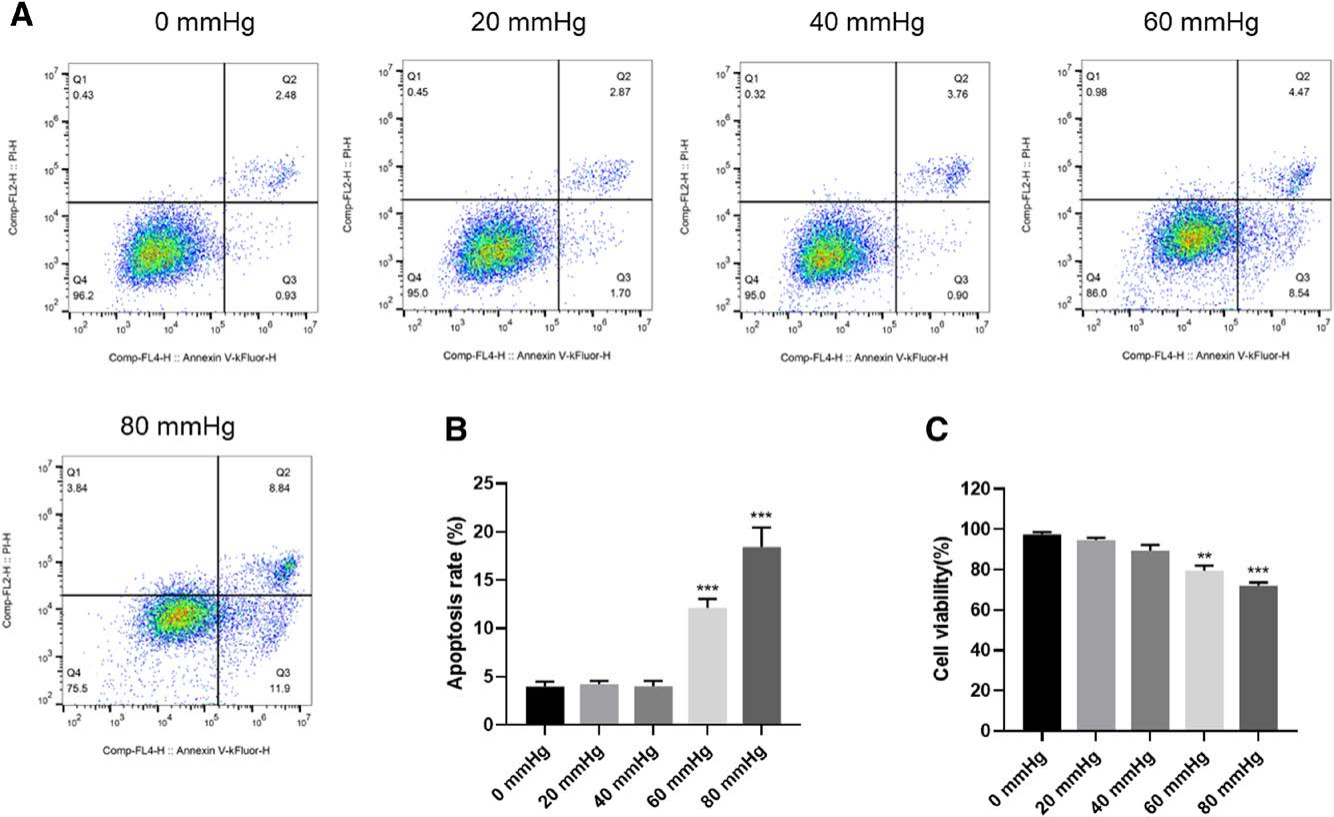
Pressurization-induced apoptosis and inhibited proliferation of RGCs; (A) apoptosis detected by flow cytometry; (B) apoptosis rate of cells in each group; (C) cell proliferation detected by CCK-8. 0 to 80 mm Hg is the value of the pressure applied to the RGCs; data are expressed as mean (standard deviation) (n = 3). One-way analysis of variance (ANOVA) was used to compare across multiple groups. **P < .01, vs the 0 mm Hg group; ***P < .001, vs the 0 mm Hg group. CCK-8 = Cell Counting Kit-8, RGCs = retinal ganglion cell.

RGCs were exposed to different pressure levels (0, 20, 40, 60, and 80 mm Hg) in a pressure-controlled culture system for 24 hours. Apoptosis was assessed by flow cytometry, and cell proliferation was measured using CCK-8 assay. In comparison to the control group, the group subjected to 60 and 80 mm Hg pressure treatment had significantly (*P* < .001) higher rate of apoptosis and lower proliferation (Fig. 1A and B). The group subjected to 80 mm Hg pressure (HP) had the highest apoptosis and lowest proliferation rate. Consequently, this pressure value was maintained for all subsequent experiments (Fig. 1C).

### 3.2. MMF serum inhibited the pressurization-induced apoptosis and promoted proliferation of RGCs

**Figure 2.**
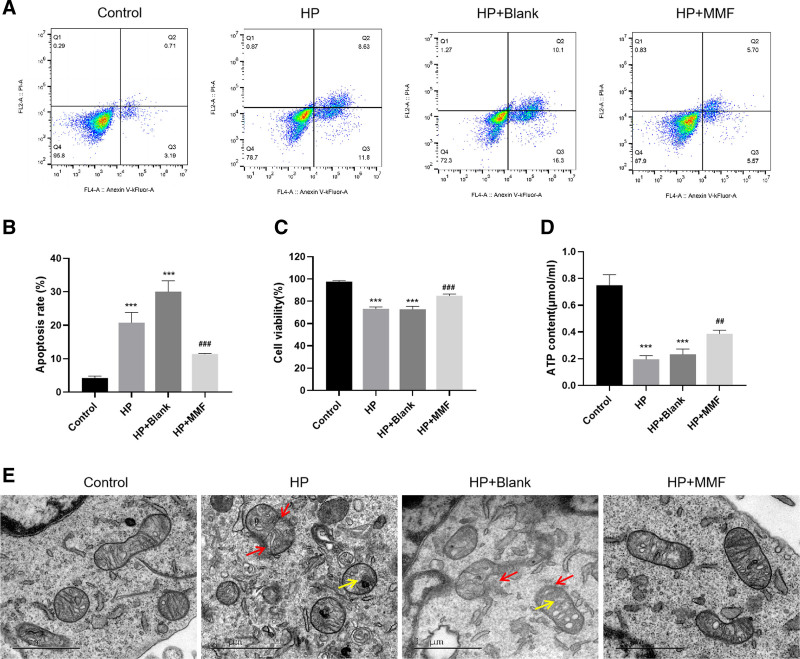
MMF serum alleviates pressurization-induced cell damage in RGCs; (A) detection of apoptosis by flow cytometry; (B) apoptosis rate in each group; (C) CCK-8 detection of cell proliferation in each group; (D) detection of ATP content; (E) observation of mitochondrial microstructures by transmission electron microscopy. MMF: Bushen-Huoxue-Mingmu-Formula; control: normal control group; HP: high pressure (80 mm Hg); HP + blank: high pressure and PBS treatment; HF + MMF: high pressure and MMF treatment; data are expressed as mean (standard deviation) (n = 3). One-way analysis of variance (ANOVA) was used to compare across multiple groups. ***P < .001, vs the control group; ###*P* < .001, vs the HP group. In the electron micrograph: red arrows represent the disruption of the mitochondrial double-membrane structure, and yellow arrows indicate the vacuolization of mitochondria. Scale bar: 1 μm. Magnification: 30 K. ATP = adenosine 5′-triphosphate, CCK-8 = Cell Counting Kit-8, RGCs = retinal ganglion cell.

Flow cytometry and CCK-8 assay were employed to examine apoptosis and proliferation in pressure-induced RGCs following exposure to 80 mm Hg pressure and to investigate the effect of MFF. The apoptosis rate was considerably higher in HP and HP + blank groups as compared to the control group (*P* < .001). In comparison to HP group, HP + MMF group showed a significant reduction in the rate of apoptosis (*P* < .001). Furthermore, HP + MMF group had a substantially higher proliferation activity (*P* < .001) (Fig. 2A–C). Thus, MMF inhibited pressurization-induced apoptosis and promoted proliferation of RGCs.

### 3.3. MMF serum alleviated pressurization-induced mitochondrial damage in RGCs

The precise function of the mitochondrion relies on its structural integrity. Herein, we examined the impact of MMF on the mitochondrial function and structure by analyzing the ATP content and microstructure of mitochondria from RGCs. MMP serum reversed the pressurization-induced decrease in the ATP content of RGCs and promoted the release of ATP (*P* < .001) (Fig. 2D). TEM images showed that the double-membrane structure of the mitochondrion was intact and mitochondrial cristae were evenly spread out in the control group. In HP and HP + blank groups, the mitochondrial double-membrane structure was disrupted, which led to severe vacuolization of the mitochondria and a decrease in the number of mitochondrial cristae. It is noteworthy that the mitochondrial morphology was improved after treatment with MMF serum (Fig. 2E). Therefore, one might propose that MMF assists in relieving glaucoma by restoring the structure and functions of mitochondria in RGCs.

### 3.4. MMF alleviated the expression of apoptosis- and mitochondrial autophagy-related proteins in RGCs

**Figure 3.**
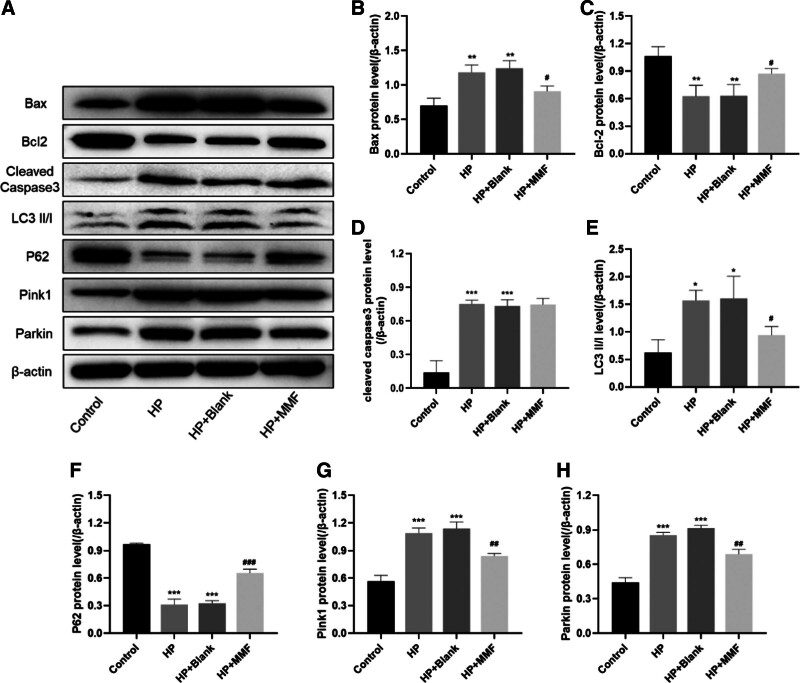
Effect of MMF serum on apoptosis and mitochondrial autophagy-related protein expression in RGCs induced by pressurization. (A) Expression levels of apoptosis and mitochondrial autophagy-related protein expression by WB; (B) Bax; (C) Bcl-2; (D) cleaved-caspase3; (E) LC3 II/I; (F) p62; (G) Pink1; (H) Parkin. Data are expressed as mean (standard deviation) (n = 3). One-way analysis of variance (ANOVA) was used to compare across multiple groups. ***P < .001, vs the control group; ##*P* < .01, vs the HP group; ###*P* < .001, vs the HP group. Bax = BCL2-associated X, Bcl-2 = B-cell lymphoma-2, LC3 = microtubule-associated proteins light chain 3, MMF = Bushen-Huoxue-Mingmu-Formula, RGCs = retinal ganglion cell

We investigated the impact of MMF serum intervention on apoptosis- and mitochondrial autophagy-related protein expression in RGCs. The levels of apoptosis-related proteins BCL2-associated X (Bax), B cell lymphoma 2 (Bcl-2), and cleaved caspase-3, and mitochondrial autophagy proteins microtubule-associated proteins light chain 3 (LC3) II/I, p62, Pink1, and Parkin were determined by Western blotting. The results showed that in comparison with the control group, HP and HP + blank groups showed significantly higher protein levels of Bax (*P* < .01), cleaved caspase-3 (*P* < .001), LC3 II/I (*P* < .05), Pink1 (*P* < .001), and Parkin (*P* < .001) along with significantly decreased levels of Bcl-2 (*P* < .01) and p62 (*P* < .001). However, MMF treatment significantly decreased the expression levels of Bax (*P* < .05), LC3 II/I (*P* < .05), Pink1 (*P* < .01), and Parkin (*P* < .01) and increased the levels of Bcl-2 (*P* < .05) and p62 (*P* < .001) (Fig. 3A–H).

### 3.5. Pink1 expression downregulation inhibited pressurization-induced apoptosis and promoted proliferation of RGCs

**Figure 4.**
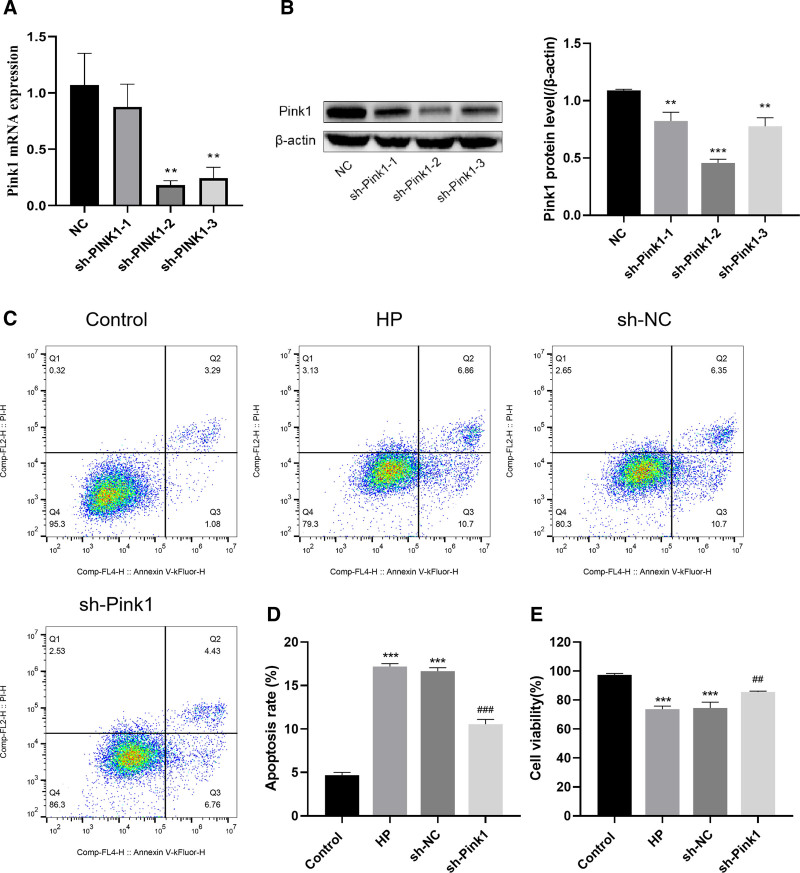
Pressurization induces apoptosis and inhibits proliferation of RGCs. (A) qRT-PCR to verify transfection of sh-Pink; (B) Western Blot to verify transfection of sh-Pink1; (C) flow cytometry to detect apoptosis; (D) apoptosis rate of cells in each group; (E) CCK-8 to detect cell proliferation in each group. Data are expressed as mean (standard deviation) (n = 3). One-way analysis of variance (ANOVA) was used to compare across multiple groups. ***P < .001, vs the NC group; ##*P* < .01, vs the HP group; ###*P* < .001, vs the HP group. CCK-8 = Cell Counting Kit-8, qRT-PCR = quantitative real-time polymerase chain reaction, RGCs = retinal ganglion cell.

Previous research has shown that pressurization activates the PINK1/Parkin pathway in RGCs. To determine whether Pink1 expression inhibition reverses the damage induced by pressure in RGCs, we performed RNA interference experiments. Three Pink1 shRNAs (sh-Pink1-1, sh-Pink1-2, and sh-Pink1-3) were designed and manufactured, and used to transfect RGCs. Their transfection efficacy was evaluated. The findings indicated a substantial decrease in the expression levels of Pink1 mRNA and protein following Pink1 shRNA transfection (*P* < .01). In particular, the expression level was comparatively lower with sh-Pink1-2 (Fig. 4A and B), which was used for subsequent experiments. Pink1 expression downregulation inhibited the stress-induced apoptosis of RGCs and promoted their proliferation (*P* < .01) (Fig. 4C–E).

### 3.6. Pink1 downregulation and MMF treatment exhibited similar effects on apoptosis- and mitochondrial autophagy-related proteins in pressurization-induced RGCs

**Figure 5.**
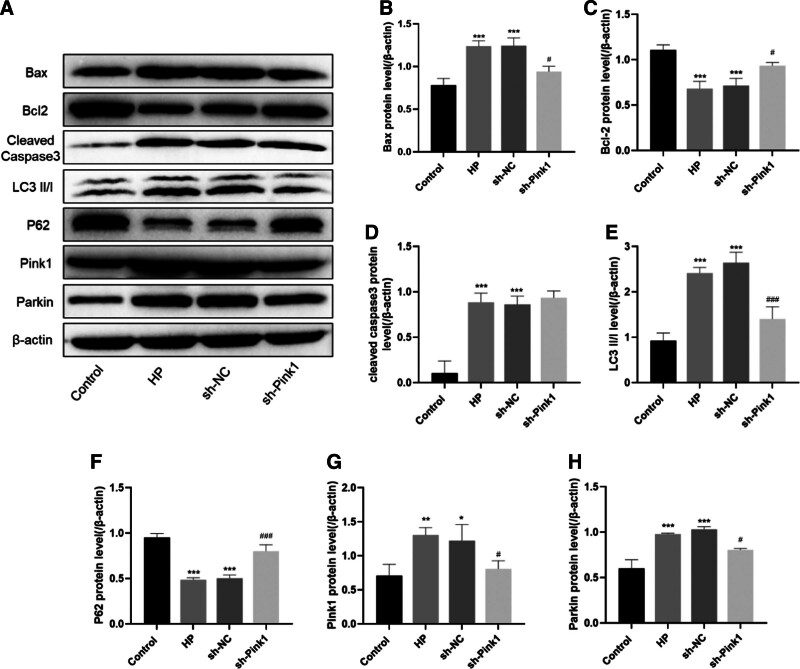
Effect of Pink1 down-regulation on stress-induced apoptosis and mitochondrial autophagy-related protein expression. (A) WB detection of apoptosis and mitochondrial autophagy-related protein expression; (B) Bax; (C) Bcl-2;(D) cleaved-caspase3; (E) LC3 II/I; (F) p62; (G) Pink1; (H) Parkin. Data are expressed as mean (standard deviation) (n = 3). One-way analysis of variance (ANOVA) was used to compare across multiple groups. ***P < .001, vs the control group; **P < .01, vs the control group; *P < .05, vs the control group; #*P* < .05, vs the HP group; ##*P* < .01, vs the HP group; ###*P* < .001, vs the HP group. Bax = BCL2-associated X, Bcl-2 = B-cell lymphoma-2, LC3 = microtubule-associated proteins light chain 3.

We explored the effects of Pink1 downregulation on the expression of apoptosis- and mitochondrial autophagy-related proteins in pressurization-treated RGCs. Pink1 expression downregulation inhibited the protein expression of Bax (*P* < .05), LC3 II/I (*P* < .001), Pink1 (*P* < .05), and Parkin (*P* < .05) and upregulated the expression of Bcl-2 (*P* < .05) and p62 (*P* < .001) proteins (Fig. 5A–H). These observations show that the downregulation of Pink1 expression and MMF treatment have similar effects in terms of alleviation of glaucoma through regulation of expression of apoptosis- and mitochondrial autophagy-related proteins.

## 4. Discussion

Glaucoma is a common blinding eye disease, characterized by progressive loss of RGCs and their axon number that leads to a gradual loss of visual field and optic nerve atrophy. Many researchers have recently directed their efforts to explore novel strategies for the treatment of glaucoma through prevention of RGC apoptosis. Traditional Chinese medicine has been shown to be effective in the treatment of glaucoma. Ligustrazine was shown to significantly reduce the apoptosis of RGCs in a rat model of IOP.^[18]^ Huo-Xue formula consisting of San Qi, Dan Shen, Yin Yang Huo (*Epimedium brevicornu Maxim*), and Ju Hua was shown to attenuate NMDA-induced loss of RGCs.^[19]^ The results of the present study reiterate that pressurization induces apoptosis and inhibits proliferation of RGCs, while MMF treatment significantly alleviates these pressurization-induced effects on RGCs. Thus, Chinese herbs that nourish the liver and kidneys, improve blood circulation, and eliminate blood stasis exhibited significant efficacy in the treatment of glaucoma. MFF preparation contains Dan Shen, San Qi, Gou Qi Zi, Ju Hua, Shu Di Huang, Jiu Yu Rou, Mu Dan Pi, Shan Yao, Fu Ling, and Ze Xie, all of which as per traditional Chinese medicine are known to exert beneficial effect on the liver and kidneys, improve blood circulation, remove blood stasis, and brighten the eyes. The active ingredients of the drugs in MFF, including salvia polyphenolates,^[20]^ Panax ginseng saponins,^[21]^ lycium barbarum polysaccharides,^[22–24]^ kaempferol,^[25]^ and paeoniflorin,^[26]^ provide the pharmacological basis for the mitigation of glaucoma.

Glaucoma is an optic neuropathy, characterized by progressive degeneration of RGCs.^[1]^ Patients with glaucoma experience RGC mortality, mainly due to structural and functional damage to mitochondria and impaired axial transport. Mitochondrial dysfunction leads to diminished expression of mitochondrial enzymes, restricted diffusion of ATP, and disturbance in the number and structure of mitochondrial autopoiesis.^[27]^ Impairment in the axial transport primarily results from IOP-induced compression, deformation, and remodeling of the sieve layer and causes mechanical harm to axons while disrupting the axonal transport of trophic factors to RGCs. Ultimately, these events culminate in the demise of RGCs.^[28–30]^ Glaucomatous RGCs exhibited an aberrant mitochondrial structure in the DBA/2J model, characterized by an increase in the quantity of mitochondria, a decrease in their size, disruption in the structure of the cristae, and augmentation in the formation of mitochondrial phagocytosis compartments within the axon.^[16]^ Our findings confirm the disruption of the mitochondrial double-membrane structure, along with severe vacuolization, reduced number of cristae, and decreased ATP content when RGCs were cultured under pressurized conditions. Thus, mitochondria were significantly damaged under pressurization. Further, the expression level of autophagy marker LC3 II/I was significantly elevated in RGCs, while that of p62 protein was significantly downregulated. The expression of apoptosis markers Bax and cleaved caspase-3 was significantly upregulated and that of Bcl-2 was significantly downregulated. Together, these changes reveal the pressurization-induced apoptosis and autophagy of RGCs. Based on these findings, we hypothesized that pressurization leads to mitochondrial structural and functional disorganization, eventually activating apoptosis and autophagy in RGCs. Notably, the treatment with MMF improved the structure and functions of mitochondria and reversed the expression of apoptosis and autophagy-related proteins. Therefore, our results confirm that MMF protected RGCs by ameliorating the mitochondrial function and suppressing apoptosis and autophagy, which may serve as the molecular basis for MMF treatment for glaucoma.

Pink1/Parkin signaling is one of the most extensively studied autophagy pathways associated with neurodegenerative diseases.^[31]^ However, its involvement in the damage caused to RGCs related to the pathogenesis of glaucoma is incompletely comprehended. As a mitochondrial enzyme, PINK1 triggers the activation and movement of Parkin from the cytoplasm to an impaired mitochondrion.^[32]^ Parkin is an E3 ubiquitin ligase located in the cytoplasm that can be triggered by PINK1.^[33]^ The depolarization of the mitochondrial membrane and engulfment of the mitochondria by autophagosomes consequently lead to the formation of mitochondrial autophagosomes in damaged mitochondria. Autophagosomes get degraded upon fusion with lysosomes. The activation of the Pink1/Parkin signaling pathway occurs throughout this process.^[34]^ We noticed a considerable upregulation in the expression of PINK1 and Parkin in RGCs after exposure to pressured culture conditions. In contrast, PINK1 and Parkin expression levels decreased after MMF treatment. Pink1 expression downregulation also significantly decreased the apoptosis of RGCs, increased their proliferation, upregulated the expression levels of the antiapoptotic protein Bcl-2, downregulated the expression of the pro-apoptotic proteins Bax and cleaved caspase-3, decreased the expression of the autophagy marker LC3 II/I, and increased the expression of the p62 protein. These results suggest that the PINK1/Parkin pathway is involved in the MMF-regulated mitochondrial autophagy process.

## 5. Conclusions

MMF attenuated mitochondrial autophagy and regulated the expression of apoptosis- and autophagy-related proteins in RGCs through inhibition of the Pink1/Parkin signaling pathway. Together these phenomena contributed to the protection of RGCs in glaucoma.

## Acknowledgments

We thank all the authors of this study, whose active participation and support in the experiments and discussions provided invaluable assistance in this study.

## Author contributions

**Conceptualization:** Qianqian Mu, Wubo Cheng.

**Data curation:** Wei Wang, Dan Zhang.

**Formal analysis:** Wei Wang, Jia Gao, Fen Yang.

**Funding acquisition:** Wubo Cheng.

**Investigation:** Fen Yang.

**Methodology:** Wei Wang, Dan Zhang.

**Project administration:** Wubo Cheng.

**Software:** Jia Gao.

**Supervision:** Qianqian Mu.

**Validation:** Fen Yang.

**Visualization:** Wei Wang.

**Writing – original draft:** Wei Wang.

**Writing – review & editing:** Qianqian Mu, Wubo Cheng.

## Supplementary Material


