# Supplementary material for: Bushen-Huoxue-Mingmu-Formula attenuates pressurization-induced retinal ganglion cell damage by reducing mitochondrial autophagy through the inhibition of the Pink1/Parkin pathway
Source: Medicine (Baltimore). 2025 Jan 10;104(2):e41257. doi: 10.1097/MD.0000000000041257 (PMC11730644; doi:10.1097/MD.0000000000041257)
Supplement: Supplementary file 1 [file medi-104-e41257-s001.docx]

Design and Synthesis of sh-Pink1

Pink1[Rat]:

NM_001106694.1 [CDS:774bp] [Protein:257aa] [5'UTR:36bp] [3'UTR:336bp] [Full:1146bp]

>NM_001106694.1|Pink1[Rat]|CDS 774bp

atggcggtgcgacaggcactgggccgaggcctgcagctgggtcgggcgctgctgctgcgcttcgctcccaagccgggcccggtgtcaggctggggcaagcccggccccggtgcggcctggggccgcggagagcgtcccggccgggtctcaagcccgggagcacagccgcgtccgctcgggctccccctcccggaccgctaccgcttcttccgccagtcggtggctgggctggcggcgcgaatccagcggcagttcgtggtgcgggcccgaggcggcgcagggccttgcggccgagcagtcttcctggccttcggactggggttggggctgatcgaggagaagcaggcggagagccggagggccgcctcggcctgtcaggagatccaggcaatttttacacagaaaaacaagcaagtgtctgacccactggacacacgacgttggcagggcttccgcctggaggattatctgataggacaggccatcggcaagggctgcaatgccgctgtgtatgaagccaccatgcccacactgccccagcacctggagaaggccaaacaccttggccttctaggaaaaggcccagatgtcgtctcaaagggagcagatggggagcaggctccaggggcccccgccttcccctttgccatcaaaatgatgtggaatatctcggcaggatcctccagcgaagccatcttaagcaaaatgagccaggagctggaagccttgggttcagcaaacaggaagggcacccttcaacagttcaggcggtag


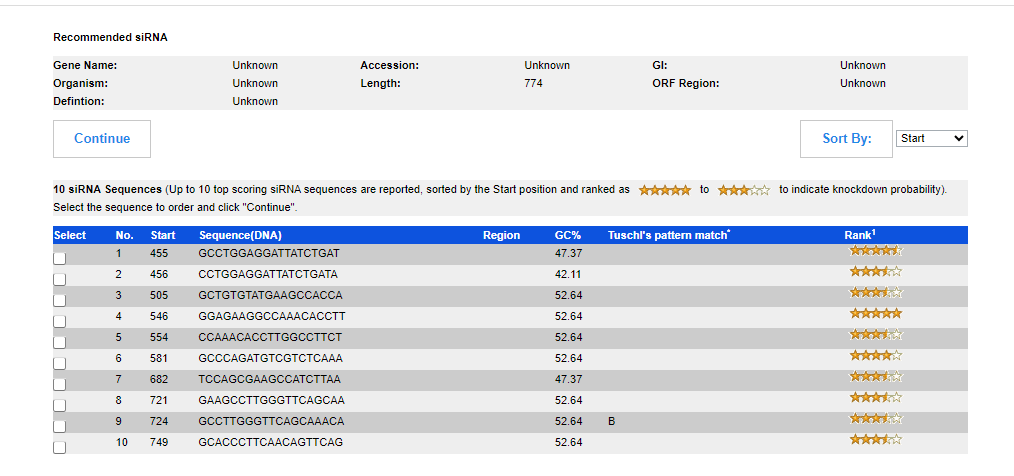


Select No. Start Sequence(DNA) Region GC% Tuschl's pattern match* Rank1

1 455 GCCTGGAGGATTATCTGAT 47.37 (sh-Pink1-1)

2 456 CCTGGAGGATTATCTGATA 42.11

3 505 GCTGTGTATGAAGCCACCA 52.64

4 546 GGAGAAGGCCAAACACCTT 52.64 (sh-Pink1-2)

5 554 CCAAACACCTTGGCCTTCT 52.64

6 581 GCCCAGATGTCGTCTCAAA 52.64 (sh-Pink1-3)

7 682 TCCAGCGAAGCCATCTTAA 47.37

8 721 GAAGCCTTGGGTTCAGCAA 52.64

9 724 GCCTTGGGTTCAGCAAACA 52.64

10 749 GCACCCTTCAACAGTTCAG 52.64
